# Anaemia secondary to critical illness: an unexplained phenomenon

**DOI:** 10.1186/2046-7648-3-4

**Published:** 2014-02-07

**Authors:** Ronan Astin, Zudin Puthucheary

**Affiliations:** 1 Department of Medicine, UCL Institute for Human Health and Performance, University College London, 4th Floor, Rockefeller Building, 21 University Street, London WC1E 6DB, UK; 2 Division of Respiratory and Critical Care Medicine, University Medicine Cluster, National University Health system, Singapore 119228, Singapore; 3 Department of Medicine, Yong Loo Lin School of Medicine, National University of Singapore, Singapore 119077, Singapore

**Keywords:** Neocytolysis, Total haemoglobin mass, Transfusion

## Abstract

Almost all patients suffering critical illness become anaemic during their time in intensive care. The cause of this anaemia and its management has been a topic of debate in critical care medicine for the last two decades. Packed red cell transfusion has an associated cost and morbidity such that decreasing the number of units transfused would be of great benefit. Our understanding of the aetiology and importance of this anaemia is improving with recent and ongoing work to establish the cause, effect and best treatment options. This review aims to describe the current literature whilst suggesting that the nature of the anaemia should be considered with reference to the time point in critical illness. Finally, we suggest that using haemoglobin concentration as a measure of oxygen-carrying capacity has limitations and that ways of measuring haemoglobin mass should be explored.

## Review

### Introduction

Critically ill patients are regularly classified by physiological syndromes such as acute respiratory distress syndrome (ARDS), multi-organ dysfunction syndrome (MODS), systemic inflammatory response syndrome (SIRS), or clinical diagnoses such as severe sepsis, acute kidney injury or traumatic brain injury. These nomenclatures hide both the heterogeneity of the patients’ presentation and the diverse diseases from which they may suffer pre-critical illness. Strikingly, despite this variation, it has been repeatedly shown that the vast majority of all patients requiring intensive care support display a falling haemoglobin concentration ([Hb]), with the majority becoming anaemic (as defined [Hb] <130 g/L in males and <120 g/L in females) [1-4]. The cause of this falling [Hb] is not clear nor its impact on patient outcomes. This review considers the time frame of anaemia of critical illness and suggests that the aetiology is related to the illness phase. The likely causes of anaemia will be discussed, and the evidence for impact on patient outcome considered. Finally, the limitations of using [Hb] as an indicator of oxygen-carrying capacity will be outlined, and the advantages of using total Hb-mass (tHb-mass) will be discussed.

#### Time course of anaemia in critical illness

Defining the temporal relationship between anaemia and critical illness is best done by dividing critical illness into three time periods:

• Pre-ICU admission

• The acute phase, covering approximately the first 7 days of ICU admission

• The chronic stage thereafter

Little is known regarding [Hb] in the lead up to ICU admission, but it is clear that on entering critical care, a large proportion of patients already have a low [Hb]. Two large studies demonstrated this; in 3,534 general admissions to ICUs in Western Europe enrolled in the ABC trial, the mean admission [Hb] was 113 ± 23 g/L and 29% had [Hb] <100 g/L [5], whilst in the 4,892 subjects enrolled to the CRIT study in general ICUs in North America, the mean baseline [Hb] was 110 ± 24 g/L [3]. A more recent study has put the proportion of patients admitted to ICU with [Hb] in the anaemic range even higher at 86% [6]. In a study of 1,023 patients admitted to 10 general ICUs in Scotland (ATICS study) each with a medical, surgical and trauma case-mix, the mean admission [Hb] was 105 g/L and 25% had [Hb] <90 g/L [7]. By combining data from multiple large studies, Walsh and Saleh have estimated that 60% to 70% of patients are anaemic on entering ICU with 20% to 30% displaying [Hb] <90 g/L [8]. However, the chronic (or non-chronic) nature of pre-ICU anaemia has not been widely studied. For the majority, though it seems unlikely to be longstanding; in both the ABC and CRIT studies, the proportion of patients entering ICU with a medical history of anaemia was only 13%.

#### The acute anaemia of critical illness

Over the first few days of admission to critical care, [Hb] continues to fall. In a study of 155 ICU patients in a general ICU, the mean admission [Hb] was 111 ± 25 g/L but by the second time point on day 7, this had fallen to 94 ± 14 g/L [2]. This is a pattern repeatedly demonstrated [6,9,10]. In the CRIT study, 44% of subjects required one or more units of packed red cells (PRC) transfused during their stay in ICU and the majority of these transfusions occurred early in their admission with a mean time to first transfusion of 2.3 ± 3.7 days (mean pre-transfusion [Hb] 86 ± 17 g/L from an admission level of 111 g/L) [3]. Chohan et al*.* showed that in a cohort of 176 patients admitted to a general ICU, 52% had [Hb] <90 g/L on day one increasing to 77% by day two [11]. Nguyen et al*.* further defined the time course of this drop by demonstrating that over the course of an ICU stay, [Hb] fell on average 5.2 ± 6.9 g/L per day, but the decline was greatest over the first 3 days when [Hb] declined by 6.6 g/L per day compared to 1.2 ± 2.9 g/L per day thereafter [4]. Importantly, these patients had no history of acute or recent blood loss, haematological disease, chronic renal dysfunction or renal replacement therapy. In this study, there was an inverse correlation between [Hb] decline and markers of illness severity (APACHEII) (*r*^2^ = 0.28, *p* < 0.001). This rapid drop in [Hb] seems then to be related to severe illness, commencing before ICU admission and continuing for a short time after.

#### Chronic anaemia in critical illness

In Nguyen’s study, there was a distinct drop-off in rate of [Hb] decline after the first 3 days. For those patients remaining in ICU >3 days, [Hb] decreased by 6.6 ± 8.4 g/L per day for the first 3 days and by 1.2 ± 2.9 g/L per day thereafter whilst for patients remaining in ICU >1 week, [Hb] dropped by 5.8 ± 6.4 g/L per day for the first 3 days and by 2.1 ± 2.3 g/L per day thereafter. This emphasizes two points: the decline in [Hb] after the acute drop slows markedly, but there is a subsequent progressive decrement in [Hb]. Describing this chronic phase in clinical studies is made difficult by blood transfusion interventions and varying transfusion practices [8]. Considering ongoing transfusion requirements alongside the associated transfusion trigger is one method of description. In a study by Corwin et al. of patients staying in ICU >7 days, 85% received one or more units of blood at some point in their stay; 40% were transfused only in the first week, but the remaining 60% of patients transfused after week one had an ongoing transfusion requirement of 2 to 3 units/week thereafter with a mean pre-transfusion haematocrit of 27% [12]. Whilst outdated phlebotomy practices accounted for some of this requirement (discussed below), more recent studies have shown a similar chronic transfusion need. In a prospective study of 100 consecutive admissions to a general ICU, the transfusion rate was 40% overall, though this climbed to 70% in those remaining in ICU >7 days [6]. In this study, the average pre-transfusion [Hb] was 73.5 ± 4.7 g/L and there was no association between transfusion and phlebotomy practices. Chant et al. reported the [Hb] variations in patients remaining in ICU for more than 30 days, excluding those with active bleeding; they found that 49% of patients received one or more PRC units after day 21 (with a mean transfusion trigger of 77 g/L), and those who received a transfusion had higher mean daily sequential organ failure assessment (SOFA) scores and requirements for organ support therapies [2]. Finally, considering [Hb] at discharge, Walsh et al*.* showed that 87% were anaemic, with 24% of men and 28% of women discharged with [Hb] <90 g/L [7] whilst in a small study of 19 critical care survivors, 53% were still anaemic 6 months after their discharge [13].

#### Aetiology of anaemia

An exhaustive discussion of the possible aetiologies is beyond the scope of this review. However, given the quite clear distinction between the acute and chronic [Hb] drop in the course of critical illness, it stands to reason that different mechanisms may be responsible for the [Hb] decline over these time periods; it is this that will be focused on.

#### Haemorrhage

In the ATICS study, of the 1,028 subjects enrolled, only 4% had a history of bleeding at ICU admission [7]. Cook et al. showed that in 1,014 mechanically ventilated patients, the incidence of clinically important gastrointestinal bleeding was 2.8% [14]. Furthermore, in Nguyen’s study, any patients with a history of recent bleeding or surgery (except where blood loss was negligible) were excluded. This suggests that the acute drop in [Hb] cannot be explained by haemorrhage. In contrast, a proportion of the chronic anaemia can be explained by blood loss events; in the ATICS study, clinically significant haemorrhage occurred in 21% of ICU patients and accounted for 40% of all transfusion episodes [15]. However, of all the patients transfused in this observational study, 54.7% received transfusions that were not associated with clinically significant haemorrhage emphasizing the importance of other causes of chronic anaemia in the critically ill.

#### Phlebotomy

Studies in the mid 1980s demonstrated the link between phlebotomy in critical care and transfusion requirement [16]. Then, being admitted to ICU increased blood sampling from a mean of 1.1 times a day to 3.4 times a day (or four times a day if there was an indwelling arterial catheter) incurring a mean blood volume loss of 41.5 ml per day [17] potentially accounting for the development of anaemia. Since the introduction of conservative phlebotomy techniques, it has been shown that though the average number of phlebotomies has not changed (mean of 3.5 ± 1.04 (SD) per patient per day), the mean blood loss can be reduced to between 8 and 22 ml per day [2,6,18], and with this, the acute drop in [Hb] over the first 3 days is independent of phlebotomy [6].

In those that remain in ICU >7 days, evidence suggests that ongoing phlebotomy may contribute significantly to anaemia. Chant et al. showed that after day 21, the number of units of RBCs transfused was significantly and independently associated with daily phlebotomy volume even when adjusted for other confounders [2].

#### Fluid status and haemodilution

**Figure 1:**
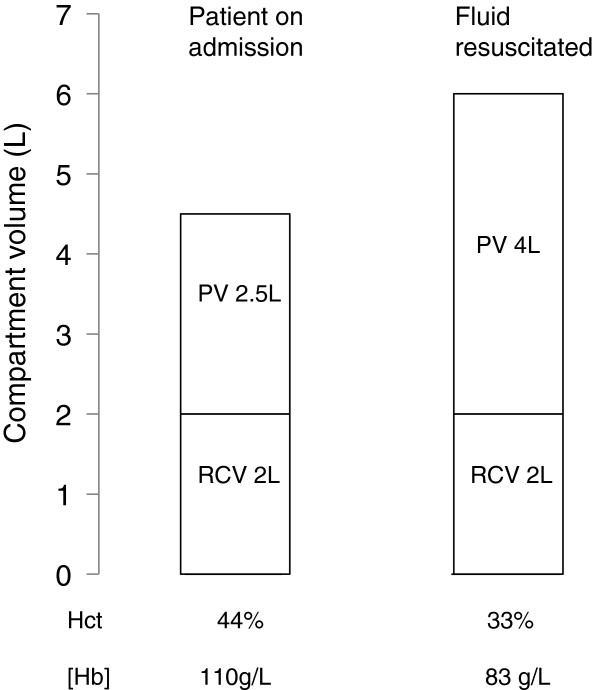
**The effect of haemodilution on full blood count parameters.** Fluid resuscitation increases the plasma volume (PV) without increasing red cell volume (RCV), resulting in a drop in Hb concentration.

Haemodilution occurs when plasma volume is expanded with no change in Hb-mass, resulting in an increased volume of distribution and consequently a decreased [Hb]. On admission to ICU, the volaemic status of the patient is assessed and addressed to maintain appropriate circulatory volume and pressure. It is often necessary to give intravascular fluid in a continuous infusion or bolus form, consequently haemodiluting the patient (Figure 1). The current literature does not allow us to attribute acute anaemia to haemodilution; most studies have not accurately measured or accounted for plasma volume in their analysis. Of those that included a measure of fluid status, Nguyen et al*.* found that fluid balance during the first 3 days did not significantly influence the fall in [Hb] [4] (though fluid status was recorded only as 24-h fluid balance). Fully accounting for haemodilution in the acute anaemia in ICU will remain a challenge whilst [Hb] is used as the marker of oxygen-carrying capacity. The advantages of using tHb-mass instead will be discussed later in the article.

#### Erythropoietin deficiency

Erythropoietin (EPO) is the major hormone regulating new red cell production in the bone marrow by inhibiting apoptosis of erythrocytic progenitor cells whilst also slowing the turnover of mature red blood cells in the reticuloendothelial system. A deficiency in EPO therefore leads to a fall in Hb. Whilst the absolute EPO levels in critical illness are not decreased [19], there is evidence for a blunted EPO response in critically ill septic patients [9,20-23]. The likely mechanism is the inhibition of the EPO gene response elements by cytokines (IL-1, IL-6, TNFα) [24,25] which are known to be increased in sepsis [26,27]. This contributes to the chronic anaemia of critical illness (in much the same way as in the anaemia of chronic disease) [28], but it cannot account for the acute drop in [Hb] as the effect of a fall in EPO (absolute or relative) on progenitor cell maturation would only be reflected in a falling [Hb] after approximately 10 days [29].

#### Haemopoietic nutrient deficiency

Efficient erythropoiesis requires not only sufficient EPO but also the availability of haemopoietic nutrients. There is evidence that in a proportion of critical ill patients, there is a deficiency in B12, folate and iron either in absolute or functional terms. In a study by Rodriguez et al*.* in non-bleeding, non-haemolysing medical ICU patients, on day 2 or 3 of ICU admission, 13% were found to have nutritional deficiencies that could contribute to anaemia (9% iron deficiency, 2% B12 deficiency, 2% folate deficiency) [30]. The burden of nutrient deficiency may even be higher than this due to the difficulty in assessing iron status during critical illness [31,32]; as an acute phase protein, ferritin is usually raised in critically ill patients without a change in total body ferritin [33] meaning that a ‘normal’ ferritin may actually reflect a total body deficit. In this way, the anaemia of critical illness resembles that of chronic disease. There is also evidence that intestinal absorption of iron may be limited in critical illness secondary to increased hepcidin levels. Hepcidin is a protein hormone which plays an important role in iron homeostasis by influencing intestinal iron absorption and iron release into the plasma from macrophages [34]. This occurs through interaction with the ferroportin transporter [35] which is expressed by enterocytes and reticuloendothelial macrophages. Increased hepcidin levels lead to decreased intestinal iron absorption and sequestration of iron within macrophages. Inflammation and infection increase hepcidin synthesis probably through IL-6 stimulation, and there is evidence that in critical illness, hepcidin levels are elevated [36]. This may be part of an evolutionary mechanism to protect against bacterial infection since bacteria require iron to support their growth [31]. Subsequent iron deficiency may be an unintended consequence. Furthermore, the inability to incorporate iron into erythroid precursors despite the presence of adequate iron stores, termed functional iron deficiency (FID), may result in ineffective haematopoiesis in the presence of ‘normal’ serum iron values. Patteril et al. showed that FID, as measured by red cell hypochromasia on flow cytometry, was present in 35% of 51 consecutive admissions in a medical ICU [37].

However, haemopoietic nutrient deficiency alone cannot explain the anaemia of critical illness; the prevalence of deficiency is low, in Patteril et al.’s study, there was no difference in [Hb] between FID and non-FID groups [37], and whilst nutrient insufficiency may contribute to chronic anaemia in critical illness, as with EPO insufficiency, the mechanism of decreased red cell production cannot explain the fall in [Hb] over the first few days.

#### Neocytolysis

**Figure 2:**
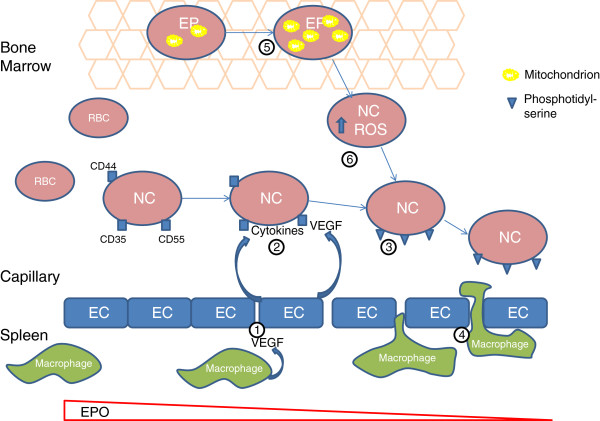
**Proposed mechanism of neocytolysis.** As the apparent concentration of EPO decreases, the permeability of the splenic endothelial cells (EC) increase (1) possibly secondary to an increase in macrophage-derived vascular endothelial growth factor (VEGF). ECs are stimulated to produce inflammatory cytokines along with VEGF (2) which cause upregulation of adhesion molecules on neocyte (NC) outer membranes and expression of phosphatidylserine (3). Macrophage processes interact with the neocytes and initiate eryptosis (4). Alternatively, erythroid progenitors (EP) in the bone marrow exposed to inflammatory conditions increase mitochondrial mass (5) which subsequently increases ROS production (6) and phosphatidylserine expression.

Neocytolysis describes the programmed destruction of new red cells (neocytes). It is a mechanism first described in astronauts returning to earth after space flight [38,39] and subsequently in climbers returning from high altitude [40,41]. In both situations, it is used to explain how red cell mass can be modulated over a short time frame of a few days leading to rapid reduction in [Hb]; in a study of astronauts returning from space flight, red cell mass was reduced by 10% to 15% over 7 to 10 days [42]. Although the mechanism has not been clearly defined, it appears that neocytolysis is dependent on red cell splenic endothelial cell-macrophage interaction. The likely mechanisms are summarized in Figure 2. The first is dependent on decreased serum EPO levels whilst the second involves oxidative damage in neocytes. Work by Song et al*.* in a mouse model of neocytolysis in the setting of hypoxia-induced polycythaemia demonstrated that reticulocytes exposed to hypoxic conditions during maturation have an increased mitochondrial mass but lower levels of the reactive oxygen species (ROS) scavenging enzyme, catalase [43]. This leads to oxidative damage in the mature reticulocyte and expression of phosphatidylserine on their outer membrane and subsequent splenic consumption. Given the inflammatory state common to most critical illnesses, it is likely that erythroid progenitors are exposed to oxidative stress to oxidative stress during maturation and thus might follow this pathway. No studies have yet demonstrated neocytolysis in critical illness but it would provide a plausible explanation for the acute fall in [Hb] which cannot easily be explained otherwise and may contribute to the ongoing anaemia of the chronic phase. However, to suggest that neocytolysis occurs as a programmed response to critical illness is to suggest that tHb-mass is purposefully downregulated.

#### Anaemia and outcome in critical illness

Evaluation of the effect of anaemia on outcome in critical illness is complicated by the impact of PRC transfusions. In addition to the complications applicable to all transfusion recipients (volume overload, transfusion-related lung injury (TRALI) and anaphylaxis), in the critically ill, there is evidence that transfusion of PRCs can cause immunomodulation, lead to increased incidence of nosocomial infection [44] and is associated with longer ICU stay [3] and greater mortality independent of organ dysfunction [3,5,44,45]. The more severely anaemic patients are likely to be exposed to more transfusions and associated risk, confounding the risk of anaemia alone.

The TRICC trial provides some evidence to suggest that anaemia in critical illness is not necessarily harmful, with particular reference to acute anaemia [46]. This randomized study of non-bleeding critically ill patients with a [Hb] <90 g/L within the first 3 days of ICU admission compared a liberal transfusion strategy (transfusion trigger [Hb] <100 g/L, [Hb] aim 100 to 120 g/L) to a restrictive strategy (transfusion trigger [Hb] <70 g/L, [Hb] aim 70 to 90 g/L). The result strongly suggests restricting blood transfusions to the lower trigger, with no difference in overall outcome between the groups, but a trend towards lower mortality in the restrictive group, particularly in the younger and less severely ill subgroup. The study did not use leucodepleted packed red cells and the storage age of the blood was unknown, both of which may have increased the adverse events in the liberal group. However, there is general agreement that a restrictive policy should be followed in most patients, with the exception of ischaemic cardiac cases where clarity is still required [47,48]. A smaller study conducted recently in 167 critically ill patients confirmed the lack of harm associated with a transfusion trigger of 70 g/L [10]. Outside critical illness, the same has been shown in high-risk patients undergoing hip surgery [49] and in upper gastrointestinal bleeds where a restrictive strategy significantly improved outcomes [50].There is also reason to believe that [Hb] lower than 70 g/L may be well tolerated; clinical reports of Jehovah’s Witness patients who refuse blood transfusion show mortality only increases when [Hb] fell below 50 g/L [51]. A recent meta-analysis of 19 randomized controlled trials investigating anaemia in critical illness from 1956 onwards and including 6,264 patients showed that there is no association between a lower transfusion threshold ([Hb] 60 to 80 g/L) and any significant complication or outcome measure [52].

There are several reasons why transfusion of PRCs in critical illness may not be beneficial. First, anaemia may confer beneficial effects on cardiac output and flow characteristics in the peripheral circulation. In healthy humans, the decreased viscosity that accompanies anaemia leads to decreased left ventricular outflow impedance resulting in an increase in cardiac output [53-55]. Second, anaemia is associated with decreased systemic vascular resistance [56] whilst an increased haematocrit causes an increase in viscosity and increased vascular resistance [57]. Additionally, changes to red cell rheology and function occur in critical illness with decreased deformability [58] and increased interaction with the endothelium likely contributing to microcirculatory pathology [58-61]. In this setting, anaemia may improve peripheral perfusion.

#### Oxygen delivery in critical illness

Other than treating haemorrhage, the most cited indications for the transfusion of PRC units in ICU are low [Hb] and to increase oxygen (O_2_) delivery (DO_2_) [5,15,62]. DO_2_ is determined by cardiac output (CO) and the oxygen content of the arterial blood (CaO_2_) can be expressed as:

$$DO_{2}=\mathrm{CO}\times \mathrm{Ca}O_{2}$$

CaO_2_ is dependent on the plasma concentration of Hb, the oxygen-binding capacity of Hb (usually considered to be 1.34 ml O_2_ per gram Hb) and the plasma partial pressure of O_2_ (which itself is a function of the solubility factor of oxygen at body temperature (0.23 ml l^-1^ kPa^-1^). This can be calculated as follows:

$$\begin{array}{c} \mathrm{Ca}O_{2}=\left(\left[\mathrm{Hb}\right]\times \mathrm{arterial}\;O_{2}\;\mathrm{saturation}\times 1.34\;\mathrm{ml}\;O_{2}/\mathrm{gHb}\right)\\ \;+\;\left(0.23\times \mathrm{Pa}O_{2}\right)\; \end{array}$$

Using this equation, transfusion of PRCs does increase DO_2_ in critically ill patients [63]. However, in the critically ill, this does not translate to an increase in oxygen utilization (as measured by oxygen consumption, VO_2_) in the tissues [8,64,65]. In a group of septic anaemic patients, Fernandes et al. demonstrated, by way of gastric tonometry and calorimetry, no increase in global or regional oxygen utilization following PRC transfusion [66], whilst Creteur et al. showed no consistent improvement in muscle oxygen utilization using near-infrared spectroscopy (NIRS) in all-comer ICU patients following transfusion [67].

Some authors have suggested that this discrepancy can be explained by alterations in the oxygen-carrying and delivery capacity of transfused blood as a consequence of their storage, termed the ‘storage lesion’. These changes include decreased deformability [68] and alterations in ATP and 2,3 DPG levels [69]. The effect of these changes would be decreased RBC capillary flow and decreased oxygen off-loading, which are not taken into account by the calculation of DO_2_ outlined above. However, the storage lesion is an *in vitro* phenomenon, and it is not clear whether these findings are replicated *in vivo*[70] though a clinical study investigating the effect of RBC storage on clinical outcomes is currently underway [71].

Whether there is a storage lesion or not, there is evidence that rather than oxygen delivery failure, the problem occurs in failure to utilize the oxygen delivered; increased tissue oxygenation has been shown in the bladder epithelium of a rat model of sepsis [72] whilst increased muscle tissue oxygen tension has been shown in ICU patients with systemic sepsis compared with controls [73,74]. Meanwhile, oxygen consumption (measured by VO_2max_) decreases in sepsis and correlates with severity of sepsis [75]. Mitochondria are the sites of oxygen utilization, and as such, these data point toward mitochondrial dysfunction, of which there is increasing evidence [76-79].

This has led to the theory of cellular hibernation proposed by Singer [80,81] which states that in the setting of sepsis, within the milieu of inflammatory cytokines and cellular dysfunction, maintaining mitochondrial mass and respiration in non-critical organs would result in increased ROS production (and potential harm to the cell) and would place an unnecessary energy demand on the body as a whole (in manufacturing mitochondrial proteins, regulating mitochondrial turnover, etc.). Instead, the cell enters a state akin to hibernation. In this situation, there is adaptive cell hypoxia where low pO_2_ can be tolerated due to adaptive changes in the drivers of electron transport [82-84]. This seems to be supported by evidence of early mitochondrial biogenesis being associated with survival in septic patients [78].

Overall, the extreme physiology of critical illness including adaptive cell hypoxia means that the usual employment of ‘normality’ in reference to Hb levels is unhelpful. The assumption that a normal [Hb] is required to optimize outcomes has been disproved. More work is required to identify where the transfusion threshold should lie [52].

#### tHb-mass and critical illness

In discussing the anaemia of critical illness, a major limitation is the inherent inaccuracy associated with measuring oxygen-carrying capacity by [Hb] rather than tHb-mass. Concentration is dependent on the circulatory volume. As discussed previously, the fluid status of patients entering ICU differs significantly. For any given [Hb], patients may have very different tHb-masses [85]. Table 1 demonstrates the importance of considering tHb-mass rather than [Hb] when calculating DO_2_. Considering a constant [Hb] of 75 g/L, increasing the inspired oxygen from 21% to 100% increases the CaO_2_(tHb-mass) by 16 ml. However, if we consider that despite a constant [Hb], actual tHb-mass might vary by 20%; this causes a 132-ml drop in CaO_2_(tHb-mass). Furthermore, since haemodilution occurs early in ICU admission but to varying degrees, not only the initial [Hb] but also its trend cannot be relied upon as a marker of oxygen-carrying capacity. Studies investigating indications for blood transfusion in ICU have repeatedly found that increasing oxygen-carrying capacity [46,86] and low [Hb] [5,62] are the most frequently reported reasons. It is likely therefore that a proportion of patients are being transfused unnecessarily. Given the risks associated with transfusion, any measure decreasing the number of unnecessary transfusions would be of benefit. Using tHb-mass as part of a decision tool will provide a more accurate representation of transfusion need and will allow the development of individualized patient transfusion pathways, reducing risk exposure. The most widely employed method to measure tHb-mass is the optimized carbon monoxide (CO) re-breathing method developed by Schmidt and Prommer [87], though this is not currently in use in clinical medicine. Briefly, a bolus of inhaled CO is used to ‘tag’ Hb, and % HbCO in blood is measured before and (6 and 8 min) after the bolus is delivered; using the Fick principle, the tHb-mass can then be calculated. There are obvious difficulties in adapting the CO re-breathing method of Schmidt and Prommer to the intubated and ventilated patient. Overcoming these issues would allow transfusion requirements to be more accurately judged and would yield additional information such as an accurate calculation of the circulating plasma volume - both would be extremely valuable in treating the critically ill patient.

**Table 1:** The relative contribution of [Hb], inspired oxygen and tHb-mass on oxygen-carrying capacity

	**Normal**	**Anaemic**	**Anaemic + oxygen**	**Decreased tHb-mass**
Inspired oxygen (%)	21	21	100	21
PaO_2_ (kPa)	12	12	85	85
SaO_2_ (%)	98	98	98	98
Hb concentration (g/L)	*150*	*75*	*75*	*75*
Dissolved oxygen (ml/L)	3	3	19	3
Hb bound oxygen (ml/L)	197	98	98	98
tHb-mass (g)	750	500	500	400
Total CaO_2[Hb]_ (ml/L)	*200*	*101*	*117*	*101*
Total CaO_2__(tHb-mass)_ (ml)	*988*	*660*	*676*	*528*

Adapted from SA McLellan [85] with permission. PaO_2_, arterial partial pressure of oxygen; SaO_2_, arterial oxygen saturation; tHb-mass, total haemoglobin mass; CaO_2 [Hb]_, oxygen-carrying capacity using [Hb]; CaO_2 [tHb-mass]_, oxygen-carrying capacity using tHb-mass.

## Conclusions

**Figure 3:**
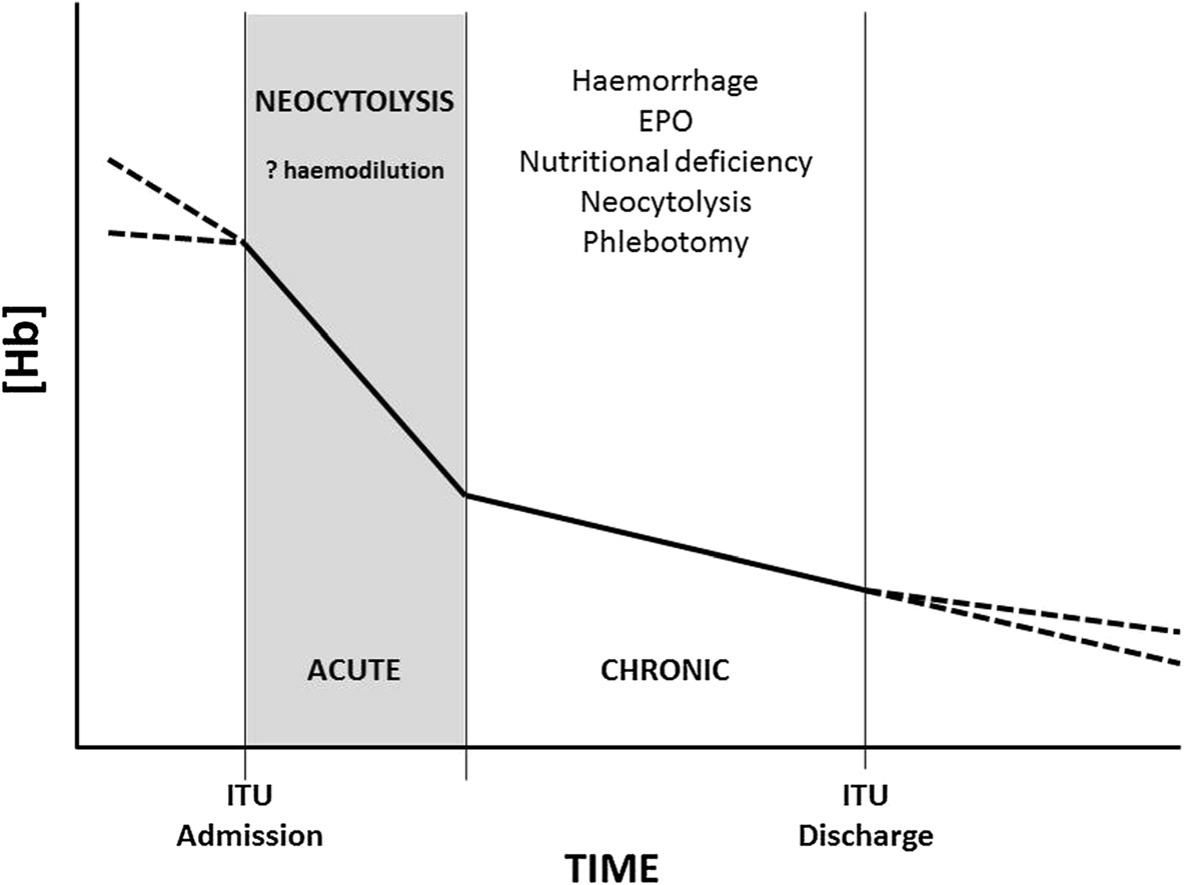
Phases of critical care anaemia.

Anaemia is ubiquitous in the critically ill population. There seems to be two distinct phases: the acute phase of the first 7 days during which the drop in [Hb] is greatest and the chronic phase where [Hb] fails to recover but does not decline at the same rate. The causes of anaemia in these phases differ (Figure 3), and most of the quoted causes may contribute to the chronic anaemia but cannot explain the acute drop in [Hb]. Neocytolysis is one mechanism by which such an acute fall in [Hb] could occur, and if the theory of cellular hibernation is considered, then a programmed reduction in Hb is logical. Neocytolysis has not yet been demonstrated in the critically ill. Finally, the development of methods to measure tHb-mass in this group is necessary if we are to more accurately assess the transfusion needs of critically ill patients.

## Abbreviations

CaO2: oxygen content of arterial blood; DO2: oxygen delivery; EPO: erythropoietin; FID: functional iron deficiency; Hb: haemoglobin; ICU: intensive care unit; IL-1: interleukin 1; IL-6: interleukin 6; NIRS: near-infrared spectroscopy; O2: oxygen; PaO2: partial pressure of oxygen in arterial blood; pO2: partial pressure of oxygen; PRC: packed red cells; ROS: reactive oxygen species; tHb-mass: total haemoglobin mass; TNFα: tumour necrosis factor alpha; VO2: oxygen consumption; VO2 max: maximal oxygen consumption.

## Competing interests

The authors declare that they have no competing interests.

## Authors’ contributions

RA and ZP contributed equally to this manuscript. Both authors read and approved the final manuscript.
